# Risk factors and traditional Chinese medicine syndromes of mild cognitive impairment in community-dwelling hypertensive older adults

**DOI:** 10.3389/fpubh.2025.1579557

**Published:** 2025-05-21

**Authors:** Jie Gao, Meirong Wu, Siyuan Jia, Yuan Tian, Dengkun Wang, Xiaojing Lei, Yongchang Diwu

**Affiliations:** ^1^The First Clinical College of Medicine, Shaanxi University of Chinese Medicine, Xianyang, China; ^2^Affiliated Hospital of Shaanxi University of Chinese Medicine, Xianyang, China; ^3^College of Basic Medicine, Shaanxi University of Chinese Medicine, Xianyang, China

**Keywords:** community, older adults, hypertension, mild cognitive impairment, influencing factors, traditional Chinese medicine syndrome elements

## Abstract

**Background:**

Hypertension is recognized as a risk factor for cognitive impairment in older adults. This study aimed to investigate the prevalence, associated factors, and traditional Chinese medicine (TCM) syndrome elements of mild cognitive impairment (MCI) among hypertensive older adults within Chinese communities.

**Methods:**

A community-based cross-sectional study was conducted involving hypertensive individuals. Participants were diagnosed with hypertension through medical history and physical examination, and were assessed for MCI using neuropsychological scales. TCM syndrome elements were evaluated using the Dementia Syndrome Scale. Logistic regression analysis was employed to identify risk factors for MCI and associated TCM syndromes.

**Results:**

The prevalence of MCI among 715 hypertensive participants was found to be 35.4%. Multivariate logistic regression analysis revealed sleep disorders as a significant risk factor for MCI (OR = 3.855, 95% CI: 2.454–6.055). Conversely, a higher education level, mental work, antihypertensive medication, and healthy lifestyle habits—including frequent tea consumption, reading, social interaction, and daily exercise—were identified as protective factors against MCI. TCM syndrome analysis indicated that marrow-deficiency, internal-heat, and phlegm-turbidity were significantly associated with an increased risk of MCI.

**Conclusion:**

The prevalence of MCI is high among older adults hypertensive individuals in Chinese communities, influenced by a combination of risk and protective factors. Sleep disorders represent a major risk factor, while modifiable lifestyle and medical factors serve as protective elements. Marrow-deficiency, internal-heat, and phlegm-turbidity are key TCM syndrome elements related to MCI. These findings underscore the importance of dynamic cognitive assessment in hypertensive patients and suggest personalized interventions that integrate both Western and TCM approaches for the prevention and management of MCI.

## 1 Introduction

Hypertension and its related complications are significant public health issues globally, affecting approximately one-third of the adult population (1). Mild cognitive impairment (MCI) is generally regarded as a transitional stage between normal cognition and dementia, characterized primarily by a slight decline in memory, thinking ability, and other cognitive functions. Statistics indicate that approximately 80% of MCI patients will eventually develop dementia (2). Research has demonstrated (3) that hypertension is a recognized, reversible, and significant risk factor for cognitive impairment, profoundly influencing the occurrence and progression of vascular dementia and Alzheimer’s disease (AD). In China, compared to individuals with normal blood pressure, the risk of developing dementia and MCI in hypertensive patients is increased by 1.86 and 1.62 times, respectively (4). Recently, the prevalence of MCI among the older adults hypertensive population has risen significantly (5). Therefore, it is imperative to conduct research on MCI within the community-dwelling hypertensive older adults.

Traditional Chinese medicine (TCM) is a comprehensive medical system with thousands of years of history. It is based on several core theories, including yin and yang, the five elements, viscera, qi, blood, body fluids, and meridians, which together form the foundation for understanding human health and disease from a holistic perspective. In traditional Chinese medicine, hypertension is often classified under disease categories such as “dizziness” and “headache,” while MCI can be categorized as related conditions like “dementia” and “forgetfulness” (6). Regarding MCI, scholars of traditional Chinese medicine from past and present generally believe that the disease is located in the brain (7), with deficiencies in internal organs and the gradual depletion of marrow identified as root causes. As deficiencies lead to excess, abnormalities in qi, blood, and body fluids occur. Pathological products such as phlegm, blood stasis, turbid toxins, and fire (heat) can obstruct the brain orifices, resulting in cognitive dysfunction, which is pivotal in the onset of dementia. However, due to the complexity of the pathogenesis, there remains a lack of widely accepted standards for syndrome differentiation in clinical Chinese medicine (8). In the prevention and treatment of hypertension with MCI, modern medicine lacks specific drugs to control disease progression. If timely intervention is not undertaken in the early stages, the patient’s cognitive condition may gradually decline, ultimately imposing a significant burden on the individual, family, and society (9). However, Chinese medicine possesses unique advantages in the prevention and treatment of MCI, characterized by its holistic approach and the principles of syndrome differentiation and treatment. The integration of traditional Chinese medicine classic prescriptions or modified prescriptions with conventional treatments can effectively prevent and manage MCI. Randomized clinical trials of Western medicine have demonstrated that it can improve cognitive function and delay cognitive decline in patients with hypertension to a certain extent (10). The concept of syndrome, which is uniquely formed based on the synthesis of classical theories and clinical experiences in traditional Chinese medicine, is guided by its theoretical framework. Syndrome elements serve as the foundation for diagnosis and treatment in traditional Chinese medicine and are essential for syndrome differentiation and precise treatment. They reflect the nature, location, and pathogenesis of the disease. Previous studies have primarily concentrated on risk factors associated with Western medicine, while research on TCM syndrome elements remains relatively limited and lacks depth. In light of this, the current study focuses on the Chinese community population. Through a cross-sectional survey, it innovatively investigates the prevalence of MCI among older adults hypertensive patients, identifies Western medicine-related risk factors, and thoroughly explores TCM syndrome elements. This aims to enhance the understanding of the pathogenesis of MCI in the context of hypertension, particularly the role of TCM syndrome, and to provide a reference for early screening, risk assessment, and more targeted prevention and treatment strategies for MCI that incorporate the characteristics of both traditional Chinese and Western medicine within the community.

## 2 Methods

### 2.1 Study design

This was a cross-sectional study. Approved by Shaanxi University of Chinese Medicine Affiliated Hospital’s Ethics Committee (SZFYIEC-YJ-2021 No. 118), this research secured written consent from all subjects.

### 2.2 Study sample

Participants included in this study were residents from nine communities in the Xi’an and Xianyang areas of China, who underwent screening in the community from March 2021 to October 2024. The screening content included anthropometric measurements, basic clinical assessments, and scale evaluations, among others. Inclusion Criteria: (1) Age range: 50–89 years; (2) The local residence duration was no less than 6 months; (3) Meet the diagnostic criteria for hypertension; (4) Signed consent confirms participant/kin agreement to study involvement; (5) The survey materials are complete and accurate. Exclusion criteria: (1) individuals who are unable to cooperate with the investigation; (2) individuals with severe vision or hearing impairment who are unable to complete the assessment; (3) patients diagnosed with secondary hypertension, dementia, schizophrenia, depression, epilepsy, encephalitis, Huntington’s disease, severe traumatic brain injury, thyroid dysfunction, liver and kidney insufficiency, malnutrition due to chronic diarrhea, and other diseases; (4) patients who require long-term medication that could potentially impact cognitive function due to underlying diseases.

### 2.3 Assessment of hypertension and MCI

The diagnosis of hypertension is based on the “Chinese Guidelines for the Prevention and Treatment of Hypertension (2024 Revised Edition),” formulated by the Chinese Guidelines for the Prevention and Treatment of Hypertension Revision Committee (11). The inclusion criteria involve a prior diagnosis of hypertension or systolic blood pressure of ≥ 140 mmHg and/or diastolic blood pressure of ≥90 mmHg, as determined during a physical examination. In this study, the determination of hypertension relied on several key factors: the individual’s self-reported medical history, blood pressure readings, and whether they were taking medication for high blood pressure. Blood pressure measurements were taken on each participant’s right arm while seated, using an electronic blood pressure monitor. Prior to measurement, participants were instructed to sit quietly for at least 5 min. The measurement was repeated at 5-min intervals, and the higher of the two readings was recorded to ensure accuracy.

The diagnosis of MCI in this study was based on the “2018 Chinese Guidelines for the Diagnosis and Treatment of Dementia and Cognitive Impairment: Diagnosis and Treatment of Mild Cognitive Impairment” and the criteria outlined in related studies (12, 13). These criteria include: (1) the patient or an informant reports cognitive impairment; (2) the Beijing version of the Montreal Cognitive Assessment (MoCA-BJ) score is less than or equal to 19 for the illiterate group, less than or equal to 22 for the primary school group, and less than or equal to 24 for the junior high school or higher group; (3) the patient maintains the ability to live independently; and (4) the patient has not yet received a diagnosis of dementia. This study utilized neuropsychological scales to comprehensively assess MCI, including the Mini-Mental Status Examination (MMSE) and the MoCA. The combination of both scales was employed to enhance the sensitivity and specificity of the results. The degree of cognitive impairment was assessed using the Clinical Dementia Rating scale (CDR), which has a scoring range of 0–3 points (14). A CDR score of ≤0.5 indicates that the criteria for dementia are not met. Activities of daily living were assessed using the Activity of Daily Living Scale (ADL), with a scoring range of 0–56 points (15). An ADL score of <22 suggests the ability for independent daily living. Depression was evaluated using the 17-Item Hamilton Rating Scale for Depression (HAMD-17), with a scoring range of 0–52 points. A score greater than 24 indicates severe depression, and this scale was used to exclude cognitive decline due to depression (16). The Hachinski Ischemic Scale (HIS) was employed to exclude vascular factors, with a score range of 0–18 points, where a score greater than 4 points suggests vascular dementia or vascular cognitive impairment (17). In summary, patients who met the criteria of positive MoCA-BJ screening, CDR ≤ 0.5 points, ADL < 22 points, HIS ≤ 4 points, and HAMD ≤ 24 points were finally diagnosed as MCI.

### 2.4 Assessment of TCM syndrome elements

The TCM syndromes of the subjects were diagnosed according to the scoring criteria of the Pattern Element Scale for Dementia (PES-D/11). This scale includes 11 syndrome elements (18): kidney-deficiency 24.5 points, spleen-deficiency 22 points, qi-deficiency 24 points, blood-deficiency 22.5 points, yin-deficiency 27 points, yang-deficiency 25.5 points, marrow-deficiency 30 points, blood-stasis 27 points, yang-hyperactivity 30 points, internal-heat 24.5 points, and phlegm-turbidity 23 points. A diagnosis of a syndrome is confirmed if the total score exceeds 7 points. The PES-D/11 scale is specifically designed for the classification and quantification of TCM dementia syndromes, including mild cognitive impairment. It effectively assesses the TCM syndrome characteristics of patients, demonstrating a sensitivity of 58.1–94.7% and a specificity of 71.2–97.3% (19). This scale serves as a reliable tool for TCM syndrome differentiation in dementia and cognitive impairment, facilitating the standardization and objectivity of clinical syndrome differentiation and treatment, and aiding clinicians in formulating individualized treatment plans.

### 2.5 Collection of other covariates

Covariates were designed independently based on relevant references (20). Other covariates collected included the sociodemographic information of the residents (age, gender, height, weight, BMI, educational level, etc.), medical history (coronary heart disease, diabetes, cerebral infarction, sleep disorders, depression, etc.), and lifestyle history (smoking history, alcohol consumption history, exercise, reading, etc.). The BMI calculation, defined as weight (kg)/height^2^ (m^2^), represents the standard equation. The medical history of diseases such as coronary heart disease, diabetes, and cerebral infarction was based on clear diagnoses from hospitals at level two or above. Sleep quality was evaluated using the Pittsburgh Sleep Quality Index (PSQI), which has a scoring range of 0–21 points. A PSQI score greater than 7 is considered indicative of the presence of a sleep disorder (21). Hypertension treatment was defined as the use of antihypertensive drugs within the past 3 months. Smoking was defined as a cumulative consumption of 100 cigarettes or more in a lifetime, while drinking was defined as consuming alcoholic beverages containing at least 1 g of pure alcohol per day for more than 1 year (22).

### 2.6 Quality control

A community screening research team was established, comprising a chief physician and six professional master’s degree students in traditional Chinese medicine, both of whom have undergone rigorous professional training. All team members received systematic training and successfully passed an assessment prior to the survey to ensure a comprehensive understanding of the questionnaire’s usage and the meaning of each item, thereby maintaining the consistency and validity of the results. The survey was conducted on-site in a one-on-one format, utilizing standardized guidance language to ensure uniformity in the survey process. Each questionnaire required approximately 20–25 min to complete. For older adults participants who faced challenges in filling out the questionnaire due to poor vision or attention, researchers provided assistance after clarifying their accurate responses, ensuring that subjective guidance was minimized. Data entry was executed using a double-entry, double-machine independent mode, with rigorous screening and verification of the collected data to ensure both accuracy and completeness.

### 2.7 Data analysis

Continuous variables were expressed as mean ± standard deviation (for normal distribution) or median (with quartiles for skewed distribution), while count data were expressed as frequency (and constituent ratio). To assess the normal distribution of continuous variables, we employed the Kolmogorov–Smirnov test. Measurement data that followed a normal distribution with homogeneous variance were analyzed using the *t*-test, whereas non-normally distributed measurement data were evaluated with the Mann–Whitney *U* test. Count data comparisons were conducted using the chi-square test or rank sum test. Influencing factors were analyzed through binary logistic regression analysis, and the Hosmer–Lemeshow test along with collinearity assessment was performed. All analyses were conducted using SPSS 26.0 software, and *p*-values less than 0.05 (two-sided) were considered statistically significant.

## 3 Results

### 3.1 General characteristics

In this study, a total of 1,790 complete datasets were collected, ultimately including 715 hypertensive patients. The average age was 69.01 ± 8.20 years, with 340 males and 375 females. Participants were partitioned into two groups according to the presence of MCI: the non-MCI and MCI groups. A total of 462 participants (64.6%) were categorized in the non-MCI group, with an average age of 69.13 ± 8.30 years. The mean MMSE score was 28 (range: 27–29), and the mean MoCA score was 26 (range: 25–27). The MCI group consisted of 253 participants, with an average age of 68.79 ± 8.01 years. The MMSE score was 25 (range: 23–27), and the MoCA score was 21 (range: 18–22). Hypertensive individuals with MCI exhibited notably reduced MMSE and MoCA scores compared to their non-MCI counterparts (*p* < 0.05).

### 3.2 Univariate analysis of influencing factors of MCI in hypertensive patients

This study conducted a univariate analysis of the sociodemographic characteristics, medical history, and lifestyle of the two groups. This study compared the sociodemographic characteristics, medical history, and lifestyle habits of the two groups. The results revealed significant differences between the two groups in various factors, including education level, employment type, place of residence, hypertension treatment, duration of hypertension, presence of diabetes mellitus, cerebral infarction, sleep disorders, tea consumption, physical activity, reading habits, computer/internet access, social frequency, and the occurrence of MCI among hypertensive older adults in the community (*p* < 0.05). The results of the analysis are presented in Table 1.

**Table 1:** Univariate analysis of influencing factors of MCI in hypertensive patients (*n* = 715).

Influencing factors	Project	Non-MCI group (*n*, %)	MCI group (*n*, %)	*χ*^2^*/Z-*value	*P-*value
Gender	Male	222(48.1)	118(46.6)	0.131^1^	0.718
	Female	240(51.9)	135(53.4)		
Age (year)	<65	154(33.3)	55(21.7)	−4.877^2^	<0.001^*^
	65–70	133(28.8)	61(24.1)		
	71–75	96(20.8)	54(21.3)		
	76–80	50(10.8)	52(20.6)		
	81–85	25(5.4)	21(8.3)		
	>85	4(0.9)	10(4.0)		
Education level	Illiterate	26(5.6)	32(12.6)	−5.318^2^	<0.001^*^
	Primary	78(16.9)	72(28.5)		
	Junior high school or higher	358(77.5)	149(58.9)		
BMI (kg/m^2^)	<18.5	10(2.2)	6(2.4)	−1.198^2^	0.231
	18.5–23.9	175(37.9)	105(41.5)		
	24.0–28.0	213(46.1)	114(45.1)		
	>28.0	64(13.9)	28(11.1)		
Living situation	Not Living Alone	416(90.0)	227(89.7)	0.018^1^	0.892
	Living Alone	46(10.0)	26(10.3)		
Working after retirement	No	450(97.4)	245(96.8)	0.192^1^	0.662
	Yes	12(2.6)	8(3.2)		
Current/past job type	Physical	310(67.1)	214(84.6)	25.530^1^	<0.001^*^
	Mental	152(32.9)	39(15.4)		
Residence	Rural	120(26.0)	84(33.2)	4.188^1^	0.041^*^
	Urban	342(74.0)	169(66.8)		
Hypertension Treatment	No	27(5.8)	26(10.3)	4.680^1^	0.031^*^
	Yes	435(94.2)	227(89.7)		
Hypertension duration (year)	<5	140(30.3)	58(22.9)	−2.507^2^	0.012^*^
	5–10	220(47.6)	122(48.2)		
	>10	102(22.1)	73(28.9)		
Coronary heart disease	No	333(72.1)	174(68.8)	0.865^1^	0.352
	Yes	129(27.9)	79(31.2)		
Diabetes	No	373(80.7)	184(72.7)	6.091^1^	0.014^*^
	Yes	89(19.3)	69(27.3)		
Hyperlipidemia	No	366(79.2)	195(77.1)	0.445^1^	0.505
	Yes	96(20.8)	58(22.9)		
Cerebral Infarction	No	376(81.4)	180(71.1)	9.911^1^	0.002^*^
	Yes	86(18.6)	73(28.9)		
Sleep disorders	No	407(88.1)	160(63.2)	61.521^1^	<0.001^*^
	Yes	55(11.9)	93(36.8)		
Smoking	No	381(82.5)	205(81.0)	0.229^1^	0.632
	Yes	81(17.5)	48(19.0)		
Drinking	No	394(85.3)	223(88.1)	1.131^1^	0.288
	Yes	68(14.7)	30(11.9)		
Staple food	Mainly wheat-based	306(66.2)	155(61.3)	3.528^1^	0.317
	Mainly rice-based	39(8.4)	18(7.1)		
	Mainly grains	22(4.8)	17(6.7)		
	Mixed rice and wheat	95(20.6)	63(24.9)		
Fruit consumption	Every day	223(48.3)	115(45.5)	−0.797^2^	0.425
	Often	13(2.8)	5(2.0)		
	Occasionally	208(45.0)	123(48.6)		
	Rarely	18(3.9)	10(4.0)		
Vegetable consumption	Every day	433(93.7)	229(90.5)	−1.543^2^	0.123
	Often	3(0.6)	4(1.6)		
	Occasionally	23(5.0)	18(7.1)		
	Rarely	3(0.6)	2(0.8)		
Tea consumption	Every day	102(22.1)	40(15.8)	−2.938^2^	0.003^*^
	Often	71(15.4)	27(10.7)		
	Occasionally	3(0.6)	1(0.4)		
	Rarely	286(61.9)	185(73.1)		
Physical exercise	Every day	382(82.7)	192(75.9)	−2.217^2^	0.027^*^
	Often	31(6.7)	24(9.5)		
	Occasionally	19(4.1)	10(4.0)		
	Rarely	30(6.5)	27(10.7)		
Reading	Every day	12(2.6)	9(3.6)	−3.079^2^	0.002^*^
	Often	54(11.7)	12(4.7)		
	Occasionally	93(20.1)	38(15.0)		
	Rarely	303(65.6)	194(76.7)		
Electronic devices	Every day	52(11.3)	19(7.5)	−2.878^2^	0.004^*^
	Often	36(7.8)	24(9.5)		
	Occasionally	59(12.8)	9(3.6)		
	Rarely	315(68.2)	201(79.4)		
Chess and card games	Every day	2(0.4)	2(0.8)	−1.824^2^	0.068
	Often	70(15.2)	23(9.1)		
	Occasionally	49(10.6)	27(10.7)		
	Rarely	341(73.8)	201(79.4)		
Social interaction	Every day	29(6.3)	15(5.9)	−3.488^2^	<0.001^*^
	Often	182(39.4)	70(27.7)		
	Occasionally	120(26.0)	64(25.3)		
	Rarely	131(28.4)	104(41.1)		

^1^Represents the Chi-square (χ^2^) value, used for group comparisons of categorical variables. ^2^Represents the value from the Mann–Whitney *U* test, used for group comparisons of ordinal categorical variables. BMI, Body Mass Index. MCI, Mild Cognitive Impairment. Often: ≥1 time per week, occasionally: ≥1 time per month, Rarely: ≥1 time per year. *p*-value < 0.05 indicates statistically significant differences between groups. * indicates *p* < 0.05 compared to the Non-MCI group. The covariates listed in table are “Univariate analysis of influencing factors of MCI in hypertensive patients” to preliminarily screen variables.

### 3.3 Univariate analysis of TCM syndrome elements of MCI in hypertensive patients

The results indicated that kidney-deficiency, marrow-deficiency, internal-heat, phlegm-turbidity, and blood-stasis were statistically significant in hypertensive older adults in the community (*p* < 0.05). Details are presented in Table 2.

**Table 2:** Univariate analysis of TCM syndrome elements of MCI in hypertensive patients.

Influencing factors		Non-MCI group (*n*, %)	MCI group (*n*, %)	*χ^2^-*value	*P-*value
Kidney-deficiency	No	312(67.5)	130(51.4)	18.063	<0.001^*^
	Yes	150(32.5)	123(48.6)		
Spleen-deficiency	No	443(95.9)	235(92.9)	3.002	0.083
	Yes	19(4.1)	18(7.1)		
Qi-deficiency	No	378(81.8)	194(76.7)	2.698	0.100
	Yes	84(18.2)	59(23.3)		
Blood-deficiency	No	420(90.9)	235(92.9)	0.831	0.362
	Yes	42(9.1)	18(7.1)		
Yin-deficiency	No	392(84.8)	202(79.8)	2.915	0.088
	Yes	70(15.2)	51(20.2)		
Yang-deficiency	No	423(91.6)	225(79.8)	1.327	0.249
	Yes	39(8.4)	28(11.1)		
Marrow-deficiency	No	381(82.5)	155(61.3)	39.159	<0.001^*^
	Yes	81(17.5)	98(38.7)		
Yang-hyperactivity	No	386(83.5)	205(81.0)	0.725	0.394
	Yes	76(16.5)	48(19.0)		
Internal-heat	No	352(76.2)	173(68.4)	5.112	0.024^*^
	Yes	110(23.8)	80(31.6)		
Phlegm-turbidity	No	362(78.4)	151(59.7)	28.115	<0.001^*^
	Yes	100(21.6)	102(40.3)		
Blood-stasis	No	404(87.4)	202(79.8)	7.316	0.007^*^
	Yes	58(12.6)	51(20.2)		

Chi-square (*χ*^2^) value, used to compare the distribution differences of TCM syndrome elements between the two groups. *P*-value < 0.05 indicates statistically significant differences between groups. * indicates *p* < 0.05 compared to the Non-MCI group.

### 3.4 Binary logistic regression analysis of MCI in hypertensive patients

**Figure 1:**
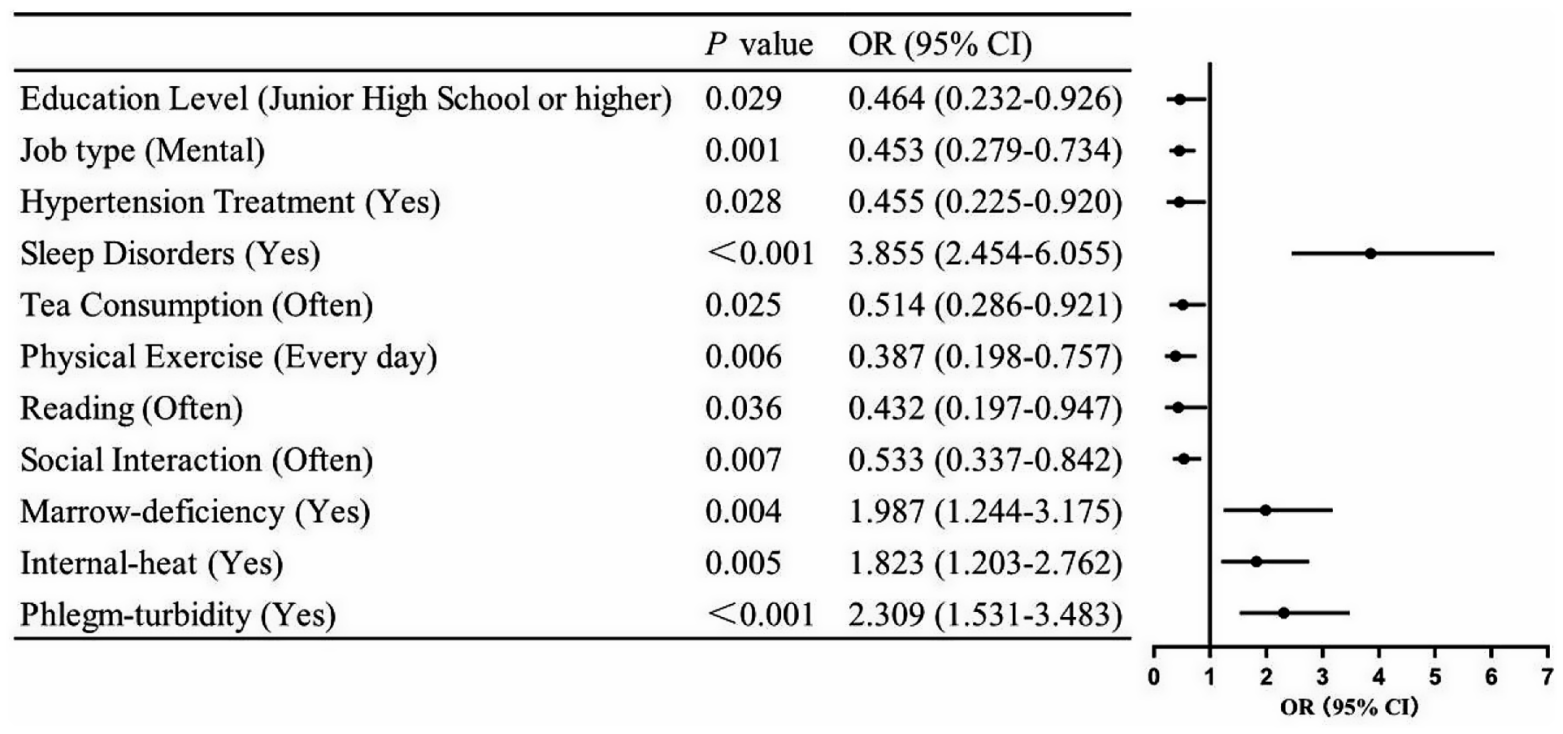
Visualization of logistic regression analysis for influencing factors of mild cognitive impairment in older adults hypertensive patients. Horizontal lines not crossing the null effect line (OR = 1) signify statistically significant influence (*p* < 0.05).

The binary variable MCI (0 = non-MCI group, 1 = MCI group) served as the outcome indicator, with statistically significant variables from the univariate analysis included as covariates in the binary logistic regression model for further examination. The specific names and values of the covariates are presented in Table 3, while the covariates controlled in the multivariate logistic regression model are detailed in Table 4. The results of the Hosmer–Lemeshow test (*χ*^2^ = 6.869, *p* = 0.551) indicated that the model exhibited a good fit, with the variance inflation factor (VIF) values ranging from 1.034 to 1.436, suggesting no significant collinearity issues among the variables. The analysis revealed that sleep disorders (OR = 3.855, 95% CI: 2.454–6.055) were risk factors for MCI among older adults hypertensive patients in the community (*p* < 0.05). Conversely, factors such as junior high school education or higher (OR = 0.464, 95% CI: 0.232–0.926), quality of mental work (OR = 0.453, 95% CI: 0.279–0.734), antihypertensive medication use (OR = 0.455, 95% CI: 0.225–0.920), frequent tea consumption (OR = 0.514, 95% CI: 0.286–0.921), reading (OR = 0.432, 95% CI: 0.197–0.947), social interaction (OR = 0.533, 95% CI: 0.337–0.842), and daily exercise (OR = 0.387, 95% CI: 0.198–0.757) were identified as protective factors against MCI in community-dwelling older adults hypertensive individuals (*p* < 0.05). Additionally, marrow-deficiency (OR = 1.987, 95% CI: 1.244–3.175), internal-heat (OR = 1.823, 95% CI: 1.203–2.762), and phlegm-turbidity (OR = 2.309, 95% CI: 1.531–3.483) were recognized as risk factors for MCI in this population (*p* < 0.05). The visualization results of the logistic regression analysis are illustrated in Figure 1.

**Table 3:** Assignment of independent variables.

Variable	Value assignment	Variable	Value assignment
Age (years)	“<65” = 1; “65 ~ 70” = 2; “71 ~ 75” = 3; “76 ~ 80” = 4; “81 ~ 85” = 5; “>85” = 6	Physical exercise	“Every day” = 1; “Often” = 2; “Occasionally” = 3; “Rarely” = 4
Education level	“Illiterate” = 1; “Primary” = 2; “Junior high school or higher” = 3	Reading	“Every day” = 1; “Often” = 2; “Occasionally” = 3; “Rarely” = 4
Job type	“Physical” = 0; “Mental” = 1	Electronic devices	“Every day” = 1; “Often” = 2; “Occasionally” = 3; “Rarely” = 4
Residence location	“Rural” = 0; “Urban” = 1	Social interaction	“Every day” = 1; “Often” = 2; “Occasionally” = 3; “Rarely” = 4
Hypertension treatment	No = 0; Yes = 1	Kidney-deficiency	No = 0; Yes = 1
Hypertension duration (years)	“<5” = 1; “5 ~ 10” = 2; “>10” = 3	Marrow-deficiency	No = 0; Yes = 1
Sleep disorders	No = 0; Yes = 1	Internal-heat	No = 0; Yes = 1
Diabetes	No = 0; Yes = 1	Phlegm-turbidity	No = 0; Yes = 1
Cerebral infarction	No = 0; Yes = 1	Blood-stasis	No = 0; Yes = 1
Tea consumption	“Every day” = 1; “Often” = 2; “Occasionally” = 3; “Rarely” = 4		

This table lists the assignment of values for each independent variable in the multivariate logistic regression analysis.

**Table 4:** Binary logistic regression analysis of MCI in hypertensive patients.

Variable	Coefficient	Standard error	Wald *χ^2^*	*P-*value	OR	95% CI
Age (ref: <65 years)						
65–70 years	0.110	0.262	0.177	0.674	1.116	0.668 ~ 1.865
71–75 years	0.040	0.297	0.018	0.894	1.040	0.581 ~ 1.863
76–80 years	0.643	0.329	3.825	0.050	1.903	0.999 ~ 3.615
81–85 years	0.228	0.452	0.256	0.613	1.257	0.518 ~ 3.046
>85 years	0.550	0.757	0.528	0.468	1.733	0.393 ~ 7.646
Education level (ref: Illiterate)						
Primary School	−0.541	0.380	2.024	0.155	0.582	0.276 ~ 1.227
Junior High School or higher	−0.768	0.353	4.746	0.029^*^	0.464	0.232 ~ 0.926
Job type (ref: Physical work)	−0.793	0.247	10.324	0.001^*^	0.453	0.279 ~ 0.734
Residence (ref: Rural)	0.016	0.218	0.005	0.943	1.016	0.662 ~ 1.558
Hypertension treatment (ref: No)	−0.787	0.359	4.800	0.028^*^	0.455	0.225 ~ 0.920
Duration of hypertension (ref: <5 years)						
5–10 years	0.450	0.238	3.583	0.058	1.568	0.984 ~ 2.499
>10 years	0.398	0.283	1.968	0.161	1.488	0.854 ~ 2.593
Sleep disorders (ref: No)	1.349	0.230	34.312	<0.001^*^	3.855	2.454 ~ 6.055
Diabetes (ref: No)	0.396	0.225	3.091	0.079	1.486	0.956 ~ 2.309
Cerebral infarction (ref: No)	0.179	0.227	0.626	0.429	1.196	0.767 ~ 1.866
Tea consumption (ref: Rarely)						
Every day	−0.270	0.255	1.121	0.290	0.764	0.464 ~ 1.258
Often	−0.666	0.298	4.996	0.025^*^	0.514	0.286 ~ 0.921
Occasionally	−0.258	1.284	0.041	0.840	0.772	0.062 ~ 9.559
Physical exercise (ref: Rarely)						
Every day	−0.948	0.342	7.705	0.006^*^	0.387	0.198 ~ 0.757
Often	−0.221	0.458	0.233	0.629	0.802	0.327 ~ 1.968
Occasionally	−0.533	0.551	0.936	0.333	0.587	0.199 ~ 1.729
Reading (ref: Rarely)						
Every day	0.769	0.587	1.719	0.190	2.157	0.683 ~ 6.810
Often	−0.839	0.400	4.395	0.036^*^	0.432	0.197 ~ 0.947
Occasionally	−0.176	0.263	0.447	0.504	0.839	0.501 ~ 1.404
Electronic devices (ref: Rarely)						
Every day	−0.605	0.378	2.571	0.109	0.546	0.260 ~ 1.144
Often	0.545	0.346	2.481	0.115	1.724	0.875 ~ 3.397
Occasionally	−0.806	0.417	3.743	0.053	0.447	0.197 ~ 1.011
Social interaction (ref: Rarely)						
Every day	−0.304	0.488	0.387	0.534	0.738	0.284 ~ 1.920
Often	−0.630	0.234	7.263	0.007^*^	0.533	0.337 ~ 0.842
Occasionally	−0.231	0.246	0.882	0.348	0.794	0.490 ~ 1.286
Kidney-deficiency (ref: No)	0.366	0.202	3.269	0.071	1.442	0.970 ~ 2.145
Marrow-deficiency (ref: No)	0.687	0.239	8.261	0.004^*^	1.987	1.244 ~ 3.175
Internal-heat (ref: No)	0.601	0.212	8.030	0.005^*^	1.823	1.203 ~ 2.762
phlegm-turbidity (ref: No)	0.837	0.210	15.917	<0.001^*^	2.309	1.531 ~ 3.483
Blood-stasis (ref: No)	0.228	0.261	0.761	0.383	1.256	0.753 ~ 2.093
Constant	−0.985	0.740	1.733	0.183	0.373	–

This table presents the results of the binary logistic regression analysis with mild cognitive impairment (MCI) as the dependent variable and statistically significant variables from the univariate analysis as independent variables. “ref” indicates the reference category. OR, Odds Ratio; 95% CI, 95% Confidence Interval. * indicates that the influence of the factor on MCI is statistically significant. Independent: age, education level, job type, residence location, hypertension treatment, hypertension duration, sleep disorders, diabetes, cerebral infarction, tea consumption, physical exercise, reading, electronic devices, social interaction, kidney-deficiency, marrow-deficiency, internal-heat, phlegm-turbidity, blood-stasis. All independent variables in the model were adjusted for each other.

## 4 Discussion

### 4.1 Analysis of the factors influencing MCI in hypertensive patients

#### 4.1.1 Sociodemographic factors

This study found that the prevalence of MCI among hypertensive older adults in the Xianyang community of Xi’an was 35.4%. Higher education levels and engagement in mental work are identified as protective factors against MCI, aligning with findings from previous research (23). Cognitive stimulation associated with mental work can enhance gray matter volume and synaptic connection density in the brain, improve compensatory abilities for age-related damage and neural plasticity, and mitigate the damage to brain nerve cells caused by free radicals (24). Furthermore, individuals with higher education levels tend to prioritize health management, which may indirectly safeguard cognitive function by managing cardiovascular risks. Consequently, it is essential to identify cognitive decline early in hypertensive patients and implement cognitive training to delay the onset and progression of the disease. Aging is widely recognized as a risk factor for MCI (25), however, this study did not establish such an association among hypertensive patients. A potential explanation for this finding is that, in the older adults population, mild hypertension may counteract age-related cognitive decline by enhancing cerebral blood perfusion through a mechanism known as “cerebral blood flow compensation” (26, 27). Additionally, cross-sectional studies often struggle to capture the long-term cumulative effects of aging, and the sample may not adequately represent the older adults patient population. The absence of detailed hypertension grade information may also diminish the observed negative impact of age. Therefore, this study did not identify significant age-related differences, highlighting the need for future longitudinal studies that incorporate blood pressure classification to further validate these findings.

#### 4.1.2 Disease factors

This study demonstrated that hypertensive patients with sleep disorders exhibit the highest risk of MCI. This finding aligns with previous research (28), which indicates that sleep disorders may adversely affect memory, cognition, and emotional regulation by altering the density and morphology of dendritic spines, as well as disrupting the secretion and clearance of *β*-amyloid protein (Aβ) (29, 30). In contrast, good sleep quality can enhance the neural connectivity between the hippocampus and the neocortex (31), thereby preserving cognitive function. It is noteworthy that hypertensive patients frequently experience emotional disorders. Such non-cognitive symptoms, including insomnia, are often overlooked in the MCI stage; however, they can indirectly exacerbate cognitive decline through mechanisms such as neuroinflammation and oxidative stress. Furthermore, this study identified antihypertensive medication as a protective factor against MCI. The underlying mechanism may be linked to hypertension-related cognitive impairment, which is associated with cerebral blood flow imbalance and impaired cerebrovascular autoregulation. Actively managing blood pressure to maintain stability can help mitigate such damage (32). Consequently, clinical attention should be directed toward sleep monitoring and blood pressure management in hypertensive patients, with an emphasis on health education to facilitate early risk identification. Although prior studies have established that the combination of hypertension and diabetes constitutes a significant risk factor for cognitive impairment (33), this study did not corroborate this conclusion. Potential explanations may include biases in patient self-reporting or sample heterogeneity, necessitating further investigation in the future through the quantification of metabolic status via biochemical indicators.

#### 4.1.3 Lifestyle factors

A healthy lifestyle, which includes tea drinking, reading, socializing, and physical exercise, can significantly reduce the risk of MCI (34–36). The specific mechanisms involved are as follows: polyphenols, theanine, and other compounds found in tea exert a neuroprotective effect through their anti-oxidative and anti-inflammatory properties, as well as by regulating the glutamate transmitter system (37). Reading and socializing facilitate information acquisition and provide psychological support, stimulating neuronal activity and delaying hippocampal atrophy, thereby enhancing attention, language expression, and memory (38, 39). Regular physical exercise, such as Tai Chi and Ba Duan Jin, can increase the release of neurotrophic factors, improve brain structure and function, and reduce the risk of cardiovascular and cerebrovascular diseases (40, 41). Therefore, it is recommended that patients with hypertension cultivate healthy eating, reading, and social habits, and engage in moderate exercise while ensuring safety, to maximize the maintenance and enhancement of their cognitive function.

### 4.2 Analysis of the TCM syndrome elements associated with MCI in hypertensive patients

This study found that among the TCM syndrome elements in hypertension patients with MCI, marrow-deficiency, internal-heat, and phlegm-turbidity were identified as the primary risk factors. Out of 253 patients, only four did not exhibit distinct pattern characteristics, while 81.4% (206 cases) of the remaining 249 patients displayed two or more pattern elements. These findings provide valuable epidemiological insights for the dialectical classification of MCI. TCM posits that cognitive function, referred to as ‘spirit’, is governed by the brain, which is described as ‘the sea of marrow’ (42). The Yellow Emperor’s Classic of Internal Medicine asserts: ‘If the sea of marrow is sufficient, the ears will be sharp and the eyes will be clear; if the sea of marrow is insufficient, the brain will be dizzy and the ears will be ringing’. A sufficient supply of brain marrow is essential for sharpness of mind, normal intelligence, and memory. Conversely, a reduction in marrow can adversely affect these functions. Modern medical research has demonstrated that the volume of the bilateral hippocampus in AD patients is significantly reduced (43), aligning with the TCM concept of marrow-deficiency. The Spiritual Pivot: Sea Theory further elaborates: ‘If the sea of marrow is insufficient, the brain will be dizzy and the ears will be ringing; the legs will be sore and weak; the eyes will be unable to see; and the person will feel lethargic and sleepy’. Marrow-deficiency can also lead to dizziness, which is closely associated with increased blood pressure, underscoring the significance of marrow-deficiency in hypertension and MCI. Internal-heat is prevalent among the older adults. Heat, characterized as a yang evil, possesses an inflammatory nature that can easily disturb the mind. Over time, the dysfunction of internal organs and the circulation of qi and blood may lead to the inadequate discharge of physiological or pathological products, resulting in excessive accumulation in the body, which generates toxins that can poison the brain’s nerves and impair cognitive function. Zhu Danxi noted in his Treatise on the Depression of the State of Mind that ‘at the age of sixty or seventy, the essence and blood are exhausted (8). Although there is no serious illness, signs of internal-heat can be observed’. Phlegm-turbidity arises from impaired metabolism of water and fluids, primarily due to deficiencies in the lungs, spleen, and kidneys. This impairment affects the body’s ability to regulate water, transport moisture, and decreases transpiration and gasification, all of which can contribute to the formation of phlegm-turbidity. When phlegm becomes turbid, it obstructs the cerebral network, leading to dizziness and cognitive decline, which can ultimately progress to dementia. As noted in “The Differentiation of Syndromes: The Door of Sickness”: “Phlegm accumulates in the chest and is entrenched outside the heart, obscuring the mind and resulting in dementia.” Modern research (44) indicates that the mechanisms of endothelial inflammation and Aβ deposition induced by metabolic abnormalities, such as hyperhomocysteinemia, are analogous to the mechanisms of endogenous turbidity poison formed by the continuous accumulation of external turbidity poison in the body. This process leads to gradual dysfunction of the viscera and impairment of qi, blood, yin, and yang within the body.

In the past, the TCM syndromes of MCI mainly included kidney-deficiency, spleen-deficiency, liver-depression, blood-stasis, etc. (45). The essence in the kidney is the original basis for human growth and development, and is closely related to human birth, growth, shape, and aging. “Suwen” points out that the kidney is responsible for bone and marrow production. The essence in the kidney can not only transform marrow into brain, but also transform spirit, thus affecting cognitive function. The liver is responsible for regulating qi, regulating emotions, and maintaining the dynamic balance of qi in various organs and tissues. Continuous negative emotions can cause the liver to lose its ability to vent and cause liver-depression. “Differential Symptoms Records Forgetfulness” points out that liver qi stagnation is a cause of forgetfulness. The spleen is the source of qi and blood, which can be distributed to the organs of the whole body and provide nutritional support for the growth and development of the human body. Spleen-deficiency produces phlegm, and phlegm and deficiency combine to invade the brain vessels, leading to cognitive impairment. When people get old, the essence of the organs gradually becomes deficient, qi deficiency makes blood flow weak, blood vessels become stagnant, and blood-stasis forms; blood-stasis blocks the heart and brain, and the spirit is obscured, and the loss of clarity leads to dementia. This study did not obtain the above results, which may be due to the subjective limitations of the syndrome differentiation scale and the fact that the population included in this study was a middle-aged and older adults hypertensive group. With the increase of age, kidney-deficiency, splee-deficiency, liver-depression, and blood-stasis are natural laws, and chronic diseases such as hypertension accelerate the above process, making older adults hypertensive patients more likely to have the above symptoms. Therefore, there was no significant difference between the two groups. In addition, due to regional influences, the dietary habits of residents in Shaanxi, China are mainly pasta, salty and spicy. Overeating these foods can easily damage the spleen and stomach, and phlegm and internal-heat accumulate in the blood vessels for a long time, leading to the loss of function of the spirit, making internal-heat and phlegm the main symptoms in this study. The study of TCM syndromes of hypertension with MCI is in line with the TCM idea of “preventing before illness and preventing changes after illness,” and is easier to combine with modern medical research methods and indicators, providing a reference for the prevention and treatment of cognitive impairment.

### 4.3 Limitations

This study has several limitations: (1) The geographical scope of this study is limited, which may result in the subjects not being representative of the broader population in China; (2) Due to the cross-sectional design, the cognitive status, influencing factors, and characteristics of TCM syndromes were assessed at a single time point, allowing for correlation analysis but precluding causal inferences. Consequently, the dynamic changes associated with age, hypertension, and cognitive status may not be adequately captured; (3) The assessment of cognitive status primarily relies on neuropsychological scales and lacks objective laboratory indicators such as brain imaging (MRI, CT), cerebrospinal fluid biomarkers (Aβ, Tau protein), and blood biomarkers for auxiliary verification, which may lead to misdiagnosis or missed diagnoses; (4) The PES-D/11 scale has been primarily developed based on TCM research, with insufficient consideration given to other cultural contexts, thereby limiting its applicability across diverse cultures. Furthermore, during the cross-cultural validation process, the translation of the scale, adjustments for cultural adaptability, and the selection of validation methods may have been inadequate, resulting in restricted cross-cultural applicability of the scale. Additionally, the judgments of different TCM practitioners may vary. Although this study underscores the importance of professional training and standardized protocols, it cannot entirely eliminate the influence of subjectivity; (5) Medical history and related information were self-reported by participants, which may be subject to unavoidable biases, such as recall bias and self-reporting inaccuracies due to memory lapses or the embellishment of certain lifestyle habits to conform to social expectations. Therefore, future studies should expand the research scope and incorporate cohort designs to investigate the effects of hypertension classification, medication, and treatment duration on cognitive function.

## 5 Conclusion

This study identified sleep quality, education level, mental workload, hypertension medication, and lifestyle habits-including tea consumption, reading, social activities, and physical exercise-as key factors influencing the risk of MCI among hypertensive older adults in the community. Additionally, marrow-deficiency, phlegm-turbidity, and internal-heat are significant TCM syndrome elements associated with MCI in this population. In conclusion, prioritizing early screening, detection, and intervention for MCI in hypertensive older adults is essential to prevent further cognitive decline.

## Data Availability

The raw data supporting the conclusions of this article will be made available by the authors, without undue reservation.
